# Limiting PcV and Tribological Performance of Mechanical Seals with Hard–Hard and Soft–Hard Tribopairs Under Water Lubrication

**DOI:** 10.3390/ma19183810

**Published:** 2026-09-08

**Authors:** Xiaoming Ren, Hui Song, He Li, Yuanren Zhou, Muqing Li, Shihao Yang, Chenxi Liu, Yunxiang Lu, Xin Li, Nan Jiang, Kazuhito Nishimura

**Affiliations:** 1 School of Materials Science and Chemical Engineering, Ningbo University, Ningbo 315211, China; 2 State Key Laboratory of Advanced Marine Materials, Zhejiang Key Laboratory of Extreme-Environmental Material Surfaces and Interfaces, Ningbo Institute of Materials Technology and Engineering, Chinese Academy of Sciences, Ningbo 315201, China; 3 Qianwan Institute of CNITECH, Ningbo 315336, China; 4 Center of Materials Science and Optoelectronics Engineering, University of Chinese Academy of Sciences, Beijing 100049, China; 5 Shanghai Institute of Optics and Fine Mechanics (SIOM), Chinese Academy of Sciences, Shanghai 201800, China; 6 Hangzhou Institute of Optics and Fine Mechanics (HIOM), Hangzhou 311421, China

**Keywords:** mechanical seal, diamond coating, water lubrication, material pairing, friction and wear mechanism

## Abstract

Water-lubricated seal interfaces are particularly vulnerable to fluid-film instability, which accelerates interfacial friction and wear and compromises the reliability and service life of underwater equipment. Here, a novel graphite-microcrystalline diamond (Graphite-MCD) soft–hard seal-face tribopair was proposed and systematically benchmarked against SiC-SiC and Graphite-SiC tribopairs under high-pressure water lubrication. Their sealing capacity, friction and wear behavior, and interfacial evolution were evaluated. The polished Graphite-MCD pair achieved the highest limiting PcV (90.73 MPa·m·s^−1^) and the lowest wear rate, markedly outperforming the SiC-SiC and Graphite-SiC pairs. Multiscale interfacial characterization showed that the SiC-SiC pair failed primarily through adhesive instability after breakdown of the water film. Although the Graphite-SiC pair reduced interfacial shear resistance, sliding generated a continuous triboreaction layer accompanied by third-body wear. In contrast, the Graphite-MCD pair preserved interfacial structural integrity and suppressed the formation of an unstable continuous reaction layer and abundant abrasive debris. This behavior enabled a synergistic combination of a low-shear lubricating interface and a stiff load-bearing counterface. These results demonstrate that improving the limiting PcV of mechanical seals cannot be achieved by reducing the friction coefficient alone; rather, it requires simultaneous control of lubricity and interfacial stability. The findings provide a mechanistic basis for designing soft–hard tribopairs for high-pressure, water-lubricated mechanical seals.

## 1. Introduction

The continuing expansion of deep-sea resource development, offshore engineering, and underwater intelligent systems toward higher pressures and greater operating depths places increasingly stringent demands on mechanical seals [1,2]. These seals are required to operate for prolonged periods under coupled high-pressure, low-temperature, corrosive, and complex loading conditions, making interfacial stability a decisive factor governing sealing reliability and service life. Under high-pressure water lubrication, the seal faces are susceptible to fluid-film instability, localized frictional heating, and interfacial wear, which can rapidly degrade sealing performance and ultimately cause failure [1,2,3,4,5]. Developing water-lubricated seal-face tribopairs that combine high load-carrying capacity, low wear, and long-term stability, while clarifying their interfacial evolution and failure mechanisms, is therefore a critical scientific challenge for improving the reliability of deep-sea equipment.

The materials selected for the seal faces and the manner in which they are paired strongly affect both sealing performance and operational reliability. Based on their mechanical properties and functional roles, seal-face tribopairs can generally be classified as hard–hard or soft–hard pairs [5,6,7]. In hard–hard pairs, high-hardness and high-strength materials support the principal contact load and provide excellent load-bearing and wear resistance [7,8]. However, when the lubricating film becomes unstable or local direct contact occurs, these pairs are prone to high friction and severe interfacial damage. In soft–hard pairs, the compliance, self-lubricity, and transfer-film-forming ability of the soft material reduce interfacial shear resistance, whereas the hard counterface carries the primary load. Such combinations are therefore widely used in high-performance mechanical seals [9,10,11]. Conventional soft–hard combinations, including Graphite-SiC, rely mainly on the formation of a graphite transfer film to lower interfacial shear resistance [12]. Although this strategy provides effective friction reduction, graphite spallation, accelerated wear, and insufficient load-bearing stability remain intrinsic limitations under high loads and extended service [7,13]. Su and co-workers investigated reaction-formed graphite/SiC composites under water-lubricated conditions and showed that increasing the retained carbon content resulted in a lower and more stable friction coefficient and reduced wear, highlighting the important role of graphite content in controlling the tribological performance of graphite/SiC composites [14]. Zhao and co-workers reported that the formation, stability, and coverage of the graphite transfer film controlled the frictional performance of impregnated graphite/stainless-steel pairs, but that increasing load disrupted the film and limited long-term load capacity [11]. The construction of stable, wear-resistant interfaces using hard coatings has consequently become an important direction in seal tribology [4,15]. Chemical-vapor-deposited diamond coatings offer high hardness, high elastic modulus, and outstanding wear resistance, providing a promising route to low-friction and highly reliable seal faces [8,16,17,18]. Zhu and co-workers paired an uncoated SiC ring with a diamond-coated SiC ring and systematically evaluated the effect of CVD diamond architecture on friction and wear. A polished graded diamond coating (GGC-MCD/SMCD/NCD/UNCD) exhibited the lowest friction coefficient and best wear resistance, demonstrating that diamond coatings can improve ceramic tribopairs by increasing interfacial hardness, load capacity, and resistance to material removal [15]. Nevertheless, the synergy between graphite lubrication and diamond load support in a soft–hard tribopair remains poorly understood. In particular, under high-pressure water lubrication, the coupled effects of graphite transfer lubrication and the hard MCD counterface on interfacial evolution, wear, and limiting PcV have not been established. Water also alters wetting, interfacial film formation, and tribological responses, producing coupled failure modes that differ markedly from those under dry sliding [6,11,19,20,21,22]. Although soft materials provide compliance, vibration damping, and interfacial adaptability, their load-bearing and wear performance can be strongly affected by water [21,22]. Likewise, the hardness, roughness, and structural stability of the hard counterface directly influence damage to the soft member [4,15].

The central question addressed here is therefore how graphite-mediated transfer lubrication and MCD-based load support interact under high-pressure water lubrication, and how this interaction governs interfacial evolution, wear failure, and the limiting PcV of soft–hard seal-face tribopairs. To answer this question, the friction and wear behavior, sealing performance, and limiting PcV of SiC-SiC, Graphite-SiC, and Graphite-MCD pairs were systematically compared. Multiscale analyses of surface morphology, near-surface microstructure, and chemical state were used to identify the interfacial evolution and failure mechanisms and to clarify the synergy between the lubricating and load-bearing interfaces. The limiting PcV values of the SiC-SiC, Graphite-SiC, and Graphite-MCD pairs were 3.40, 50.19, and 90.73 MPa·m·s^−1^, respectively, while the corresponding wear rates of the rotating rings were 8.3 × 10^−10^, 9.5 × 10^−10^, and 0.15 × 10^−10^ mm^3^·N^−1^·m^−1^. The Graphite-MCD pair therefore provided the best overall performance. These findings deepen the mechanistic understanding of soft–hard tribopairs under high-pressure water lubrication and support the design of critical tribological components for deep-sea equipment, seawater pumps, and high-speed water-lubricated bearings.

## 2. Materials and Methods

### 2.1. Materials

The mechanical-seal assembly used in this study consisted of a rotating ring and a stationary stepped ring. The seal rings had an inner diameter of 40 mm and an outer diameter of 75 mm, while the stepped section of the stationary ring had an outer diameter of 64 mm and an inner diameter of 56 mm. The graphite rings were made of phenolic-resin-impregnated graphite (M106F) and were supplied by Shanghai Dongxin Sealing Co., Ltd. [Shanghai, China]. The SiC rings were made of pressureless-sintered silicon carbide with a SiC purity of 98.5% and were supplied by Ningbo Foken Technology Co., Ltd. [Ningbo, China]. The diamond suspension, containing diamond particles with a diameter of 10 μm, was supplied by Tuocheng Hongguang Diamond Technology Co., Ltd. [Shangqiu, China]. Tantalum wire with a diameter of 0.5 mm was supplied by Ningxia Orient Tantalum Industry Co., Ltd. [Shizuishan, China].

### 2.2. Preparation of the Diamond Coating

**Figure 1:**
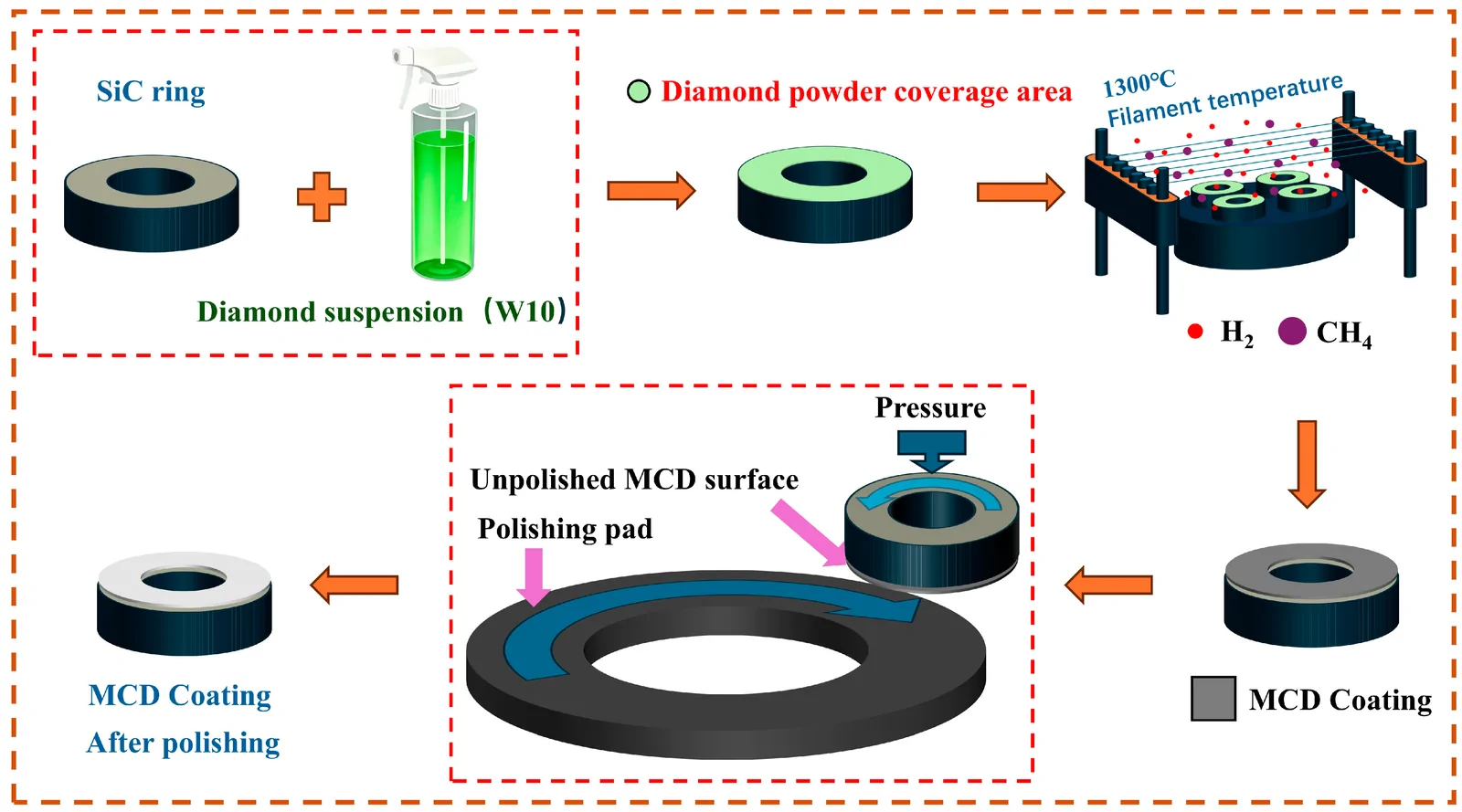
Schematic of MCD coating deposition and mechanical polishing on the SiC seal ring.

The preparation route for the polished microcrystalline diamond (MCD)-coated SiC seal ring is shown in Figure 1. A SiC rotating ring was used as the substrate, and the MCD coating was deposited by hot-filament chemical vapor deposition (HFCVD). During deposition, a CH_4_/H_2_ mixture (CH_4_:H_2_ = 45:1500 sccm) was used as the reaction gas, and tantalum wires with a diameter of 0.5 mm were employed as hot filaments. The filament-to-substrate distance was maintained at 10 mm, and the spacing between adjacent filaments was 15 mm. Each filament was powered independently at an input power of 1 kW, and the filament temperature was maintained at approximately 1300 °C during deposition. The chamber pressure was maintained at 4 kPa, and deposition was conducted for 4000 min. Under these conditions, an MCD coating with a grain size of approximately 4–8 μm was obtained. After deposition, the MCD coating was mechanically polished to reduce the surface roughness and improve its interfacial tribological performance. Polishing was performed using an SP40 system (Xiboer) [Beijing, China] under a constant load of 3.5 kg for 6 h, yielding a polished MCD seal ring with nanoscale surface roughness for subsequent friction, wear, and sealing-performance tests.

### 2.3. Evaluation of the Limiting Performance of the Seal-Face Tribopairs

Mechanical-seal performance was evaluated using a dedicated high-pressure, high-speed PcV test rig capable of reproducing mechanical-seal operation under high-load and high-speed conditions while continuously monitoring the frictional response of the sealing interface and the seal-failure process. Deionized water was used as the test medium, the chamber pressure was maintained at 0.2 MPa, and the axial load was fixed at 2000 N. The initial rotational speed was set to 200 r·min^−1^. Each speed level was maintained for 1 min, after which the rotational-speed setpoint was instantaneously increased by 100 r·min^−1^. This stepwise speed-increasing procedure was continued until seal failure occurred or the rotational speed reached 10,000 r·min^−1^. Three independent limiting PcV tests were conducted for each tribopair (n = 3). The median of the three measured limiting PcV values was used as the representative value, while the minimum and maximum values were used to define the corresponding error range. Likewise, the steady-state friction coefficients were reported with error ranges corresponding to the minimum and maximum values obtained from three independent tests (n = 3). The PcV value was calculated and monitored in real time by the test system according to the following equation:

$$PcV=P_{C}v=\frac{F_{c}}{A}\times v$$

where *Pc* is the nominal contact pressure of the sealing interface, in MPa; *Fc* is the effective closing force acting on the seal face, in N; *A* is the nominal contact area of the sealing interface, in mm^2^; and *v* is the relative sliding velocity at the stepped sealing track, in m·s^−1^. The nominal contact area was calculated according to:

$$A=\frac{\pi (D_{o}^{2}-D_{i}^{2})}{4}$$

where *D_o_* = 64 mm and *D_i_* = 56 mm are the outer and inner diameters of the stepped sealing face, respectively, giving *A* = 753.98 mm^2^. The relative sliding velocity was calculated as:

$$v=\frac{\pi d_{m}H}{60}$$

where *d_m_* = 60 mm is the mean diameter of the stepped sealing region and *N* is the instantaneous rotational speed, in r·min^−1^. The nominal contact pressure used for the limiting PcV calculation was *Pc* = 3.0723 MPa.

**Figure 2:**
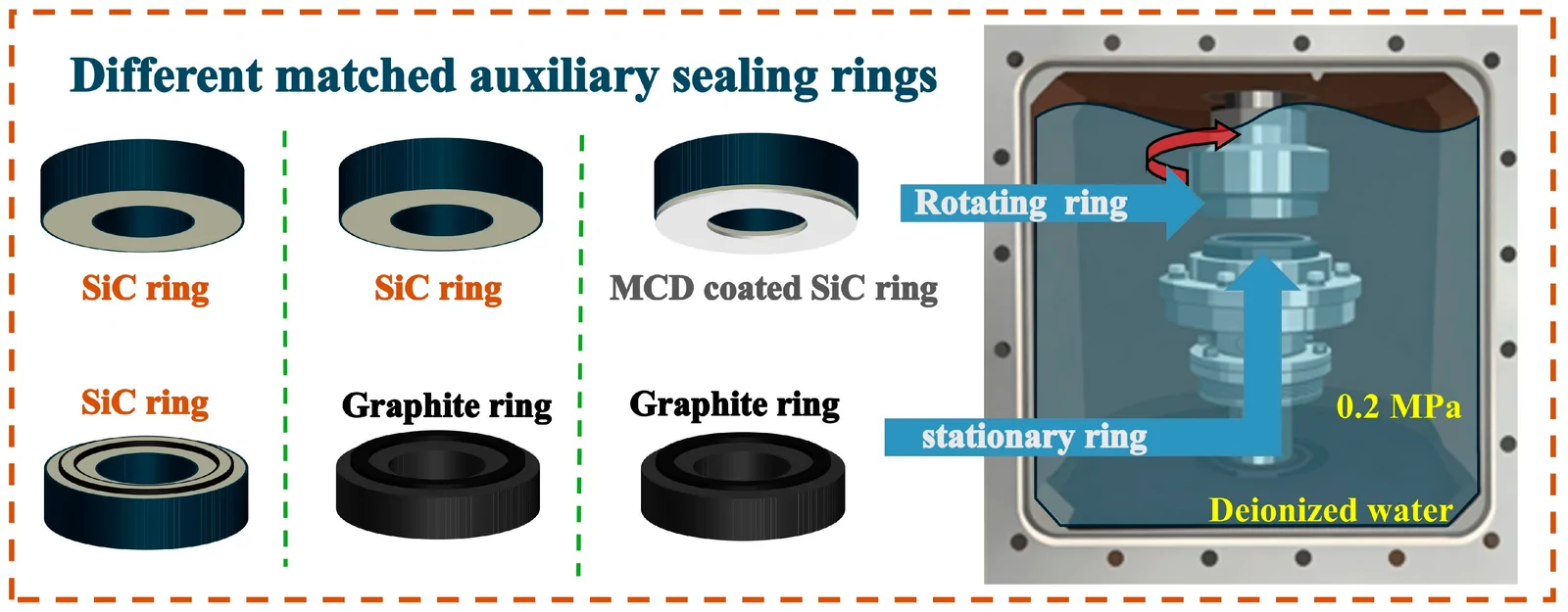
Schematic of the mechanical-seal test configurations for the SiC-SiC, Graphite-SiC, and Graphite-MCD tribopairs, the red arrow indicates the rotation direction of the moving ring.

Seal failure was defined by the occurrence of any one of the following criteria: (i) seal leakage triggering the alarm system; (ii) persistent abnormal noise during operation; or (iii) friction torque exceeding the preset threshold of 20 N·m. In addition to the limiting PcV tests, long-duration wear tests were conducted to compare the wear resistance of the different tribopairs [7]. The chamber pressure was maintained at 0.2 MPa, while the axial load and rotational speed were fixed at 500 N and 1000 r·min^−1^, respectively, and each test was conducted for 180 min. Three independent fixed-condition wear tests were performed for each tribopair under identical conditions (n = 3). For the quantitative wear results, the error bars represent the minimum-to-maximum range obtained from the three independent tests. For each independent wear test, three cross-sectional profiles were randomly selected from different positions along the wear track of the rotating ring. The median wear cross-sectional area obtained from the three profiles was multiplied by the circumference of the wear track to determine the wear volume, where the circumference was calculated using the mean diameter of the stepped sealing region (60 mm). The specific wear rate was then calculated according to the following equation:

$$W=\frac{V_{w}}{FL}$$

where *W* is the specific wear rate, in mm^3^·N^−1^·m^−1^; *V_w_* is the wear volume, in mm^3^; *F* is the applied normal load, in N; and *L* is the total sliding distance, in m. The wear volume obtained from profilometry was converted from μm^3^ to mm^3^ before calculation. The total sliding distance was calculated on the basis of the mean diameter of the stepped sealing region (60 mm), the rotational speed, and the test duration. For the stationary ring, the wear-step height was measured and used as the quantitative parameter for comparing the wear of the different tribopairs. Figure 2 illustrates the mechanical-seal testing procedure.

### 2.4. Characterization

The surface morphology of the diamond coating was examined using field-emission scanning electron microscopy (SEM; Regulus 8230, Hitachi, Tokyo, Japan). Surface chemical analysis was performed using X-ray photoelectron spectroscopy (XPS; AXIS SUPRA+, Kratos Analytical, Manchester, UK) equipped with a monochromated Al Kα X-ray source. The surface roughness and wear tracks of the diamond coating were characterized using laser confocal microscopy. Atomic force microscopy (AFM) measurements were performed using a scanning probe microscope (SPM; Bruker, Karlsruhe, Germany) in TappingMode under ambient conditions at room temperature. A silicon cantilever probe was used to scan the sample surface line by line, and the surface-height signal was recorded to reconstruct two-dimensional topographic images over a scanning area of 10 μm × 10 μm. Raman spectra of the coating before and after polishing, as well as before and after tribological testing, were acquired using a Raman spectrometer (LabRAM HR Evolution, HORIBA, Longjumeau, France) with an approximately 1 μm laser spot, an 1800-line grating, and a 532 nm excitation laser. Phase analysis was performed using an X-ray diffractometer (D8 DISCOVER, Bruker, Karlsruhe, Germany) equipped with a Cu rotating-anode X-ray source and Cu Kα radiation. The diffraction patterns were collected over a 2θ range of 10–120° at a scanning rate of approximately 0.122°·s^−1^. The morphology and near-surface structure of the sliding interfaces were further examined using high-resolution transmission electron microscopy (HRTEM; Talos, Thermo Fisher Scientific, Waltham, MA, USA).

## 3. Results and Discussion

### 3.1. Surface Morphology and Structural Characteristics of the MCD Coating Before and After Mechanical Polishing

**Figure 3:**
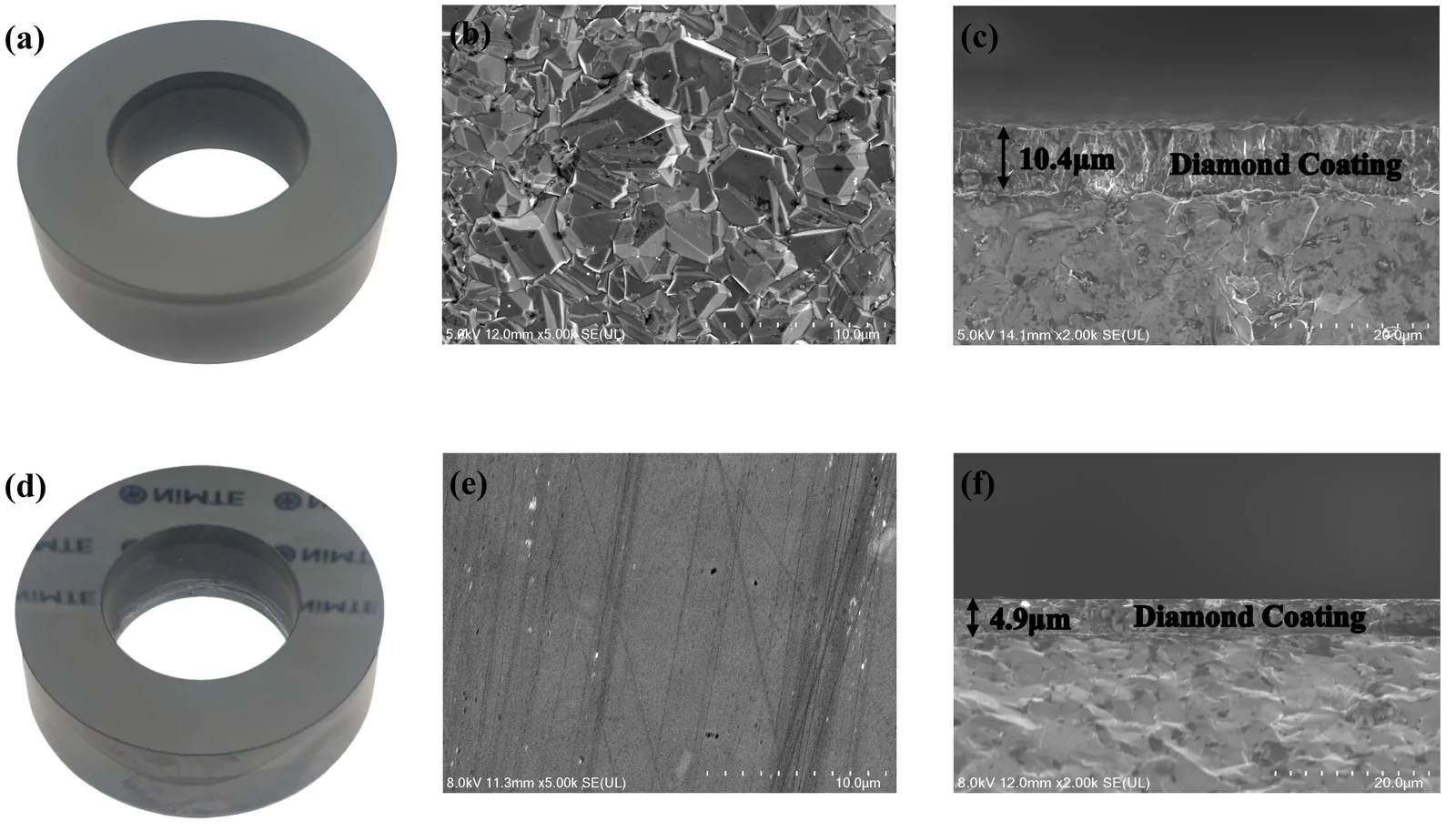
Macroscopic photographs and surface and cross-sectional SEM images of the MCD-coated SiC seal ring: (**a**–**c**) before polishing and (**d**–**f**) after polishing.

Figure 3 presents macroscopic images and surface and cross-sectional microstructures of the MCD-coated SiC seal ring before and after mechanical polishing. The as-deposited coating was continuous and dense and consisted of faceted grains approximately 4–8 μm in size. Preferential grain growth produced pronounced asperities and sharp grain edges, resulting in substantial surface relief (Figure 3b). Cross-sectional SEM revealed a typical columnar structure with a thickness of approximately 10.4 μm. The coating/substrate interface was continuous and intact, with no obvious pores, cracks, or delamination (Figure 3c), confirming the high structural integrity of the deposited MCD layer. Mechanical polishing removed the protruding grain tops and produced a much smoother surface without detectable crack propagation, localized spallation, or interfacial failure (Figure 3e). The coating thickness decreased from 10.4 to 4.9 μm (Figure 3f), indicating that planarization occurred mainly through removal of coarse surface grains and asperities from the as-deposited layer without damaging the coating body or the coating/substrate interface. During HFCVD, continuous nucleation and preferential growth generate a polycrystalline surface with substantial relief; polishing preferentially removes the highest features and progressively homogenizes the real contact surface. Importantly, this reduction in topographic variation was achieved while preserving the overall coating and interface integrity, demonstrating that material removal was confined primarily to the near-surface region. For mechanical seals, a flatter interface reduces local stress concentration and mechanical interlocking caused by grain asperities during initial contact and facilitates the establishment of a more uniform and stable water-lubricated interface. The polished MCD coating therefore provided a consistent initial contact state for evaluating the friction, wear, and limiting PcV of the different tribopairs, minimizing interference from initial roughness differences.

**Figure 4:**
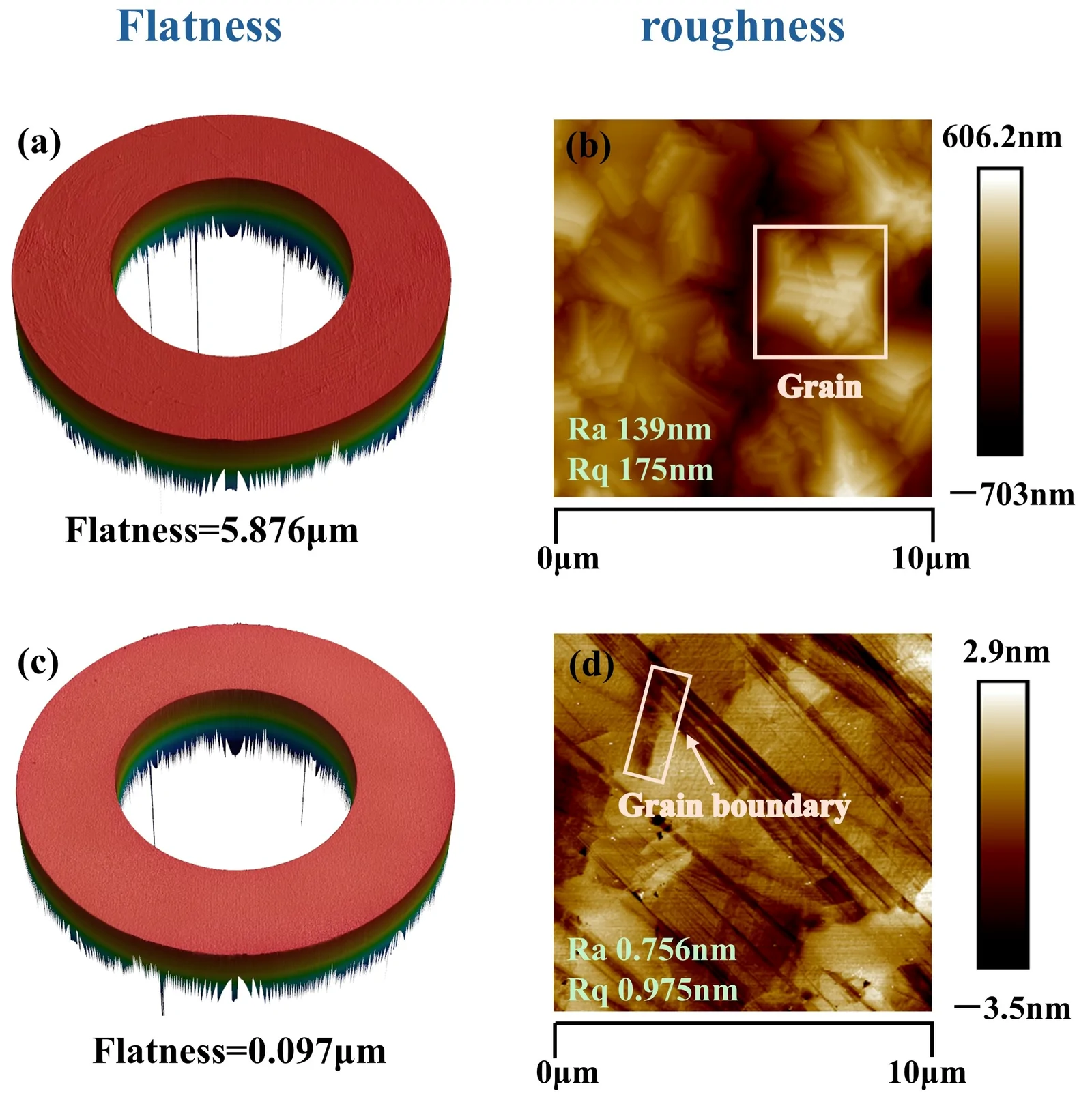
Three-dimensional profiles and AFM surface topographies of the MCD coating: (**a**,**b**) before polishing and (**c**,**d**) after polishing.

The effect of mechanical polishing on seal-face geometry and nanoscale surface morphology was quantified by three-dimensional optical profilometry and atomic force microscopy (AFM), as shown in Figure 4. Before polishing, the MCD-coated SiC ring exhibited a flatness deviation of 5.876 μm, indicating pronounced macroscopic waviness (Figure 4a). After polishing, this value decreased to 0.097 μm (Figure 4c), demonstrating a substantial improvement in geometric uniformity and overall planarity. AFM further revealed the nanoscale evolution of the surface. The as-deposited coating consisted of intact faceted grains approximately 4–8 μm in size with pronounced height differences between adjacent grains. The arithmetic mean roughness (Ra) and root-mean-square roughness (Rq) were 139 and 175 nm, respectively (Figure 4b). After polishing, the protruding grain tops were removed, the sharp edges were transformed into continuous plateau-like regions, and only a small number of machining traces remained along the polishing direction [23]. Ra and Rq decreased to 0.756 and 0.975 nm, respectively (Figure 4d). Thus, polishing simultaneously improved macroscopic flatness and nanoscale roughness, producing a highly uniform, smooth, and structurally stable surface [15]. Because seal-face flatness and roughness jointly determine the real contact area, local contact stress, and ability to form a fluid film, the polished surface is expected to promote more uniform contact, suppress mechanical interlocking, and provide consistent conditions for the formation of both a water film and a graphite transfer film. Consequently, the subsequent measurements more directly reflect the intrinsic interfacial evolution of the Graphite-MCD pair rather than differences in initial topography [3,17].

**Figure 5:**
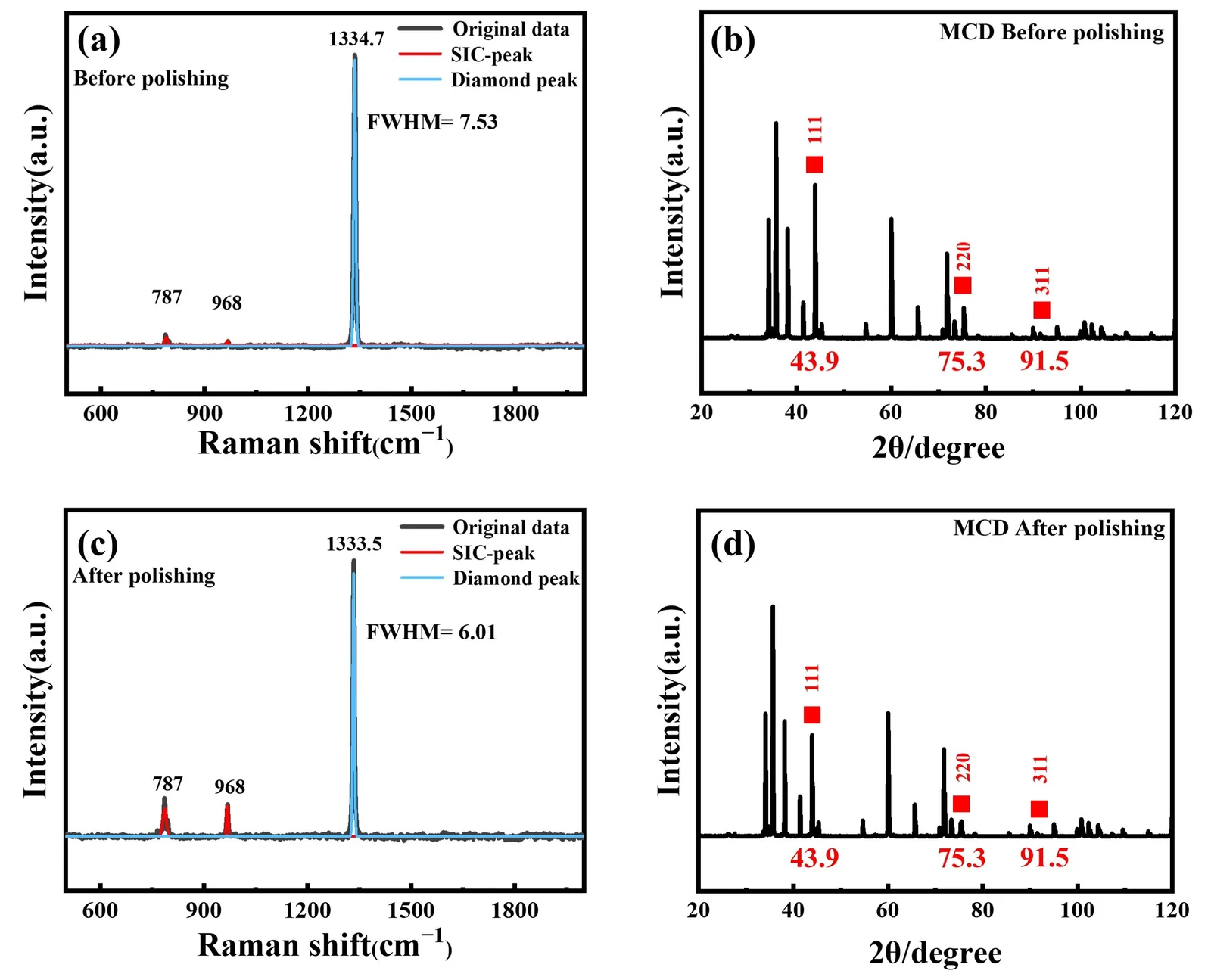
Raman spectra and XRD phase analysis of the MCD coating: (**a**,**b**) before polishing and (**c**,**d**) after polishing.

Raman spectroscopy and XRD were used to clarify the effects of mechanical polishing on the crystal structure and residual-stress state of the MCD coating (Figure 5). Before polishing, the characteristic diamond peak appeared at 1334.7 cm^−1^, blue-shifted from the stress-free diamond position at 1332 cm^−1^, indicating residual compressive stress in the coating (Figure 5a) [24]. After polishing, the peak shifted to 1333.5 cm^−1^, suggesting partial relaxation of near-surface compressive stress (Figure 5c). The full width at half maximum of the diamond peak decreased from 7.53 to 6.01 cm^−1^, indicating improved near-surface crystalline integrity after polishing. The XRD patterns before and after polishing both contained the diamond (111), (220), and (311) reflections, with essentially unchanged peak positions and relative intensities (Figure 5b,d). No graphitic carbon or other secondary phases were detected [25]. Mechanical polishing therefore did not induce a phase transformation in the MCD coating; instead, it removed surface material mechanically without causing a thermally driven structural transition. The SiC substrate bands at 787 and 968 cm^−1^ became more intense after polishing because the reduced coating thickness increased the Raman contribution from the substrate, consistent with the cross-sectional SEM observations (Figure 5a,c). Overall, polishing substantially planarized the seal face while preserving the diamond phase and crystal structure, reducing near-surface residual compressive stress, and improving crystalline integrity. The absence of detectable graphitization, secondary-phase formation, or structural degradation confirms that polishing generated a reproducible and stable interfacial state for evaluating the Graphite-MCD tribopair.

### 3.2. Tribological Performance of the Seal-Face Tribopairs Under Water Lubrication

**Figure 6:**
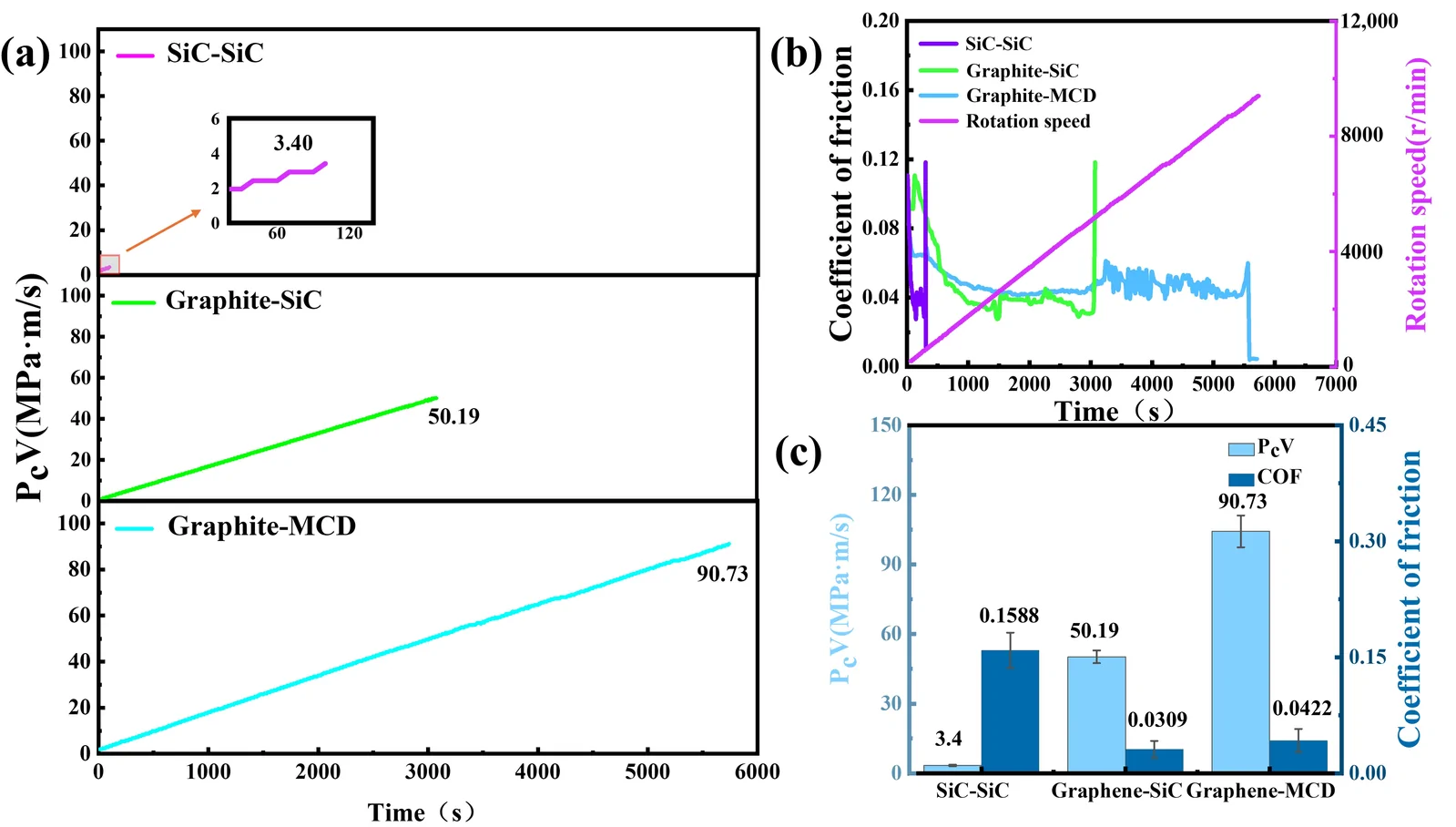
Limiting PcV performance and frictional behavior of the different seal-face tribopairs: (**a**) PcV evolution under limiting conditions; (**b**) friction coefficient and rotational speed as functions of time under limiting conditions; and (**c**) limiting PcV values and steady-state friction coefficients of the different seal-face tribopairs. Error bars in (**c**) represent the minimum-to-maximum range of three independent tests (n = 3).

To systematically compare the limiting service performance of the different seal-face tribopairs, limiting PcV tests were conducted using the mechanical-seal PcV test rig, and the results are presented in Figure 6. The three tribopairs exhibited markedly different load-carrying limits (Figure S1). The SiC–SiC pair underwent frictional instability first and exhibited a limiting PcV of only 3.40 MPa·m·s^−1^. Replacing one SiC face with graphite substantially improved the limiting performance of the Graphite–SiC pair, increasing the limiting PcV to 50.19 MPa·m·s^−1^. The Graphite–MCD pair exhibited the highest limiting PcV during the entire stepwise speed-increasing process, reaching 90.73 MPa·m·s^−1^, approximately 81% higher than that of the Graphite–SiC pair, demonstrating that the choice of seal-face materials strongly influences the limiting service capability of mechanical seals. The frictional responses further revealed distinct failure behaviors among the three tribopairs. The SiC–SiC pair developed severe friction-torque fluctuations when the rotational speed increased to approximately 300 r·min^−1^ and rapidly became unstable. The Graphite–SiC pair failed by seal leakage at 5200 r·min^−1^, corresponding to a PcV of 50.19 MPa·m·s^−1^, whereas the Graphite–MCD pair did not exhibit pronounced friction-torque fluctuations until the rotational speed reached 9400 r·min^−1^, at which point the PcV reached 90.73 MPa·m·s^−1^, indicating that it could maintain stable sealing contact under substantially higher load and speed conditions. Notably, the Graphite–SiC and Graphite–MCD pairs exhibited similar steady-state friction coefficients of approximately 0.032 and 0.041, respectively, despite their markedly different limiting PcV values, indicating that the magnitude of the average friction coefficient alone cannot directly reflect the severity of material wear. After the running-in stage, the Graphite–SiC pair exhibited a relatively low and stable coefficient of friction (COF), which may be associated with the low shear strength of graphite and the formation of a transfer layer during sliding. However, this low-friction state may have been accompanied by continuous removal and transfer of graphite material; therefore, a lower COF does not necessarily correspond to lower wear. As PcV further increased and approached the limiting condition, the COF of the Graphite–SiC pair increased abruptly from approximately 0.04 to approximately 0.11, indicating pronounced destabilization of the previously stable frictional interface. Under a constant normal load, an increase in COF corresponds to an increase in friction force, while the combination of increased friction force and higher sliding velocity enhances frictional energy dissipation at the interface, thereby promoting debris generation and material removal (Figure 6b,c). These observations demonstrate that the limiting service capability of a mechanical seal is not governed solely by the friction coefficient [16]. Overall, the three seal-face tribopairs exhibited distinctly different limiting PcV performances, suggesting that their sliding interfaces underwent different interfacial evolution processes under high-load and high-speed conditions and that these differences in interfacial evolution played a critical role in determining the limiting service performance of the corresponding seal-face tribopairs.

### 3.3. Surface Morphology and Structural Analysis of the Worn Seal Rings

**Figure 7:**
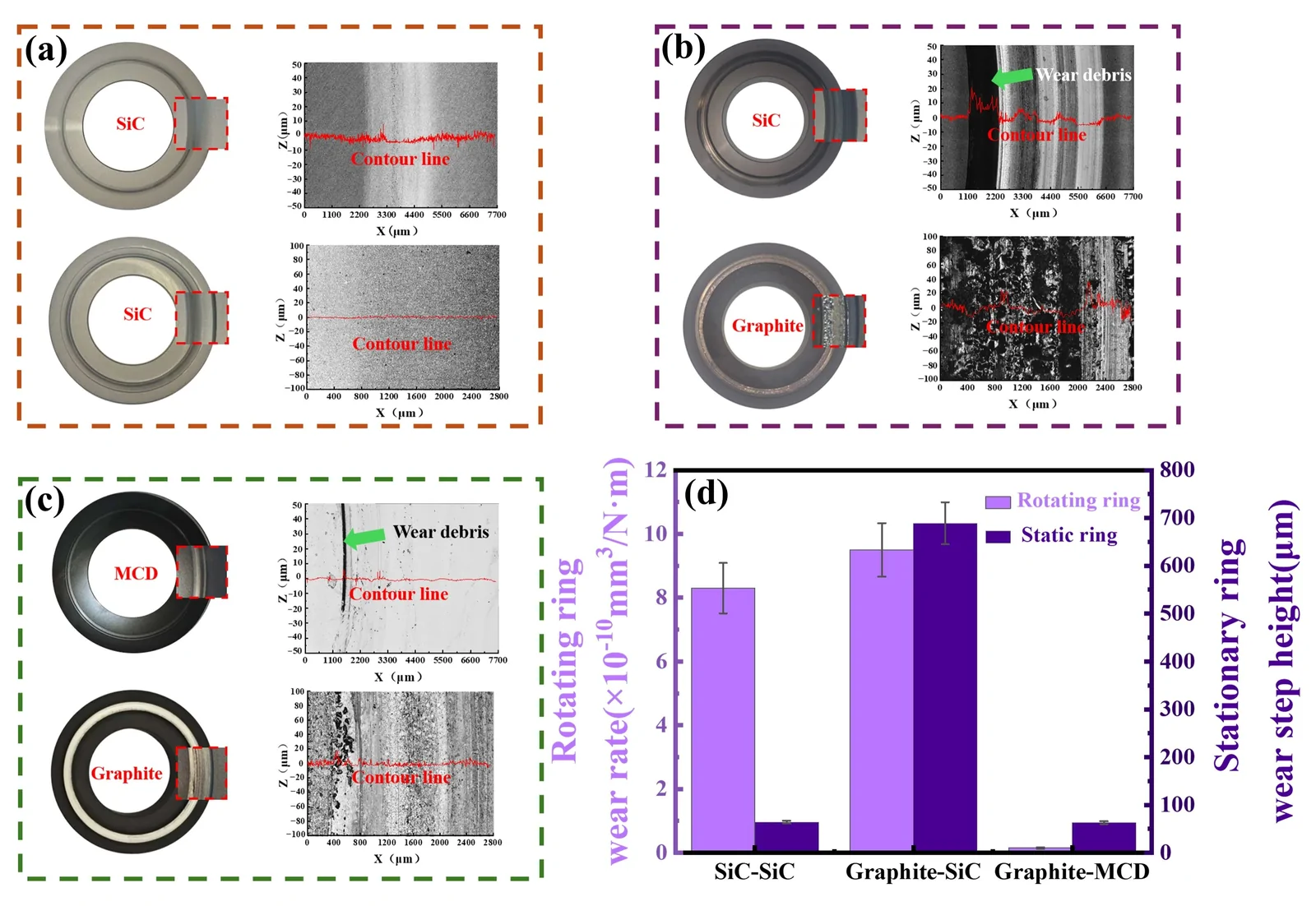
Optical microscopic images and two-dimensional wear profiles of the different seal-face tribopairs after the limiting friction tests: (**a**) SiC–SiC, (**b**) Graphite–SiC, and (**c**) Graphite–MCD; and (**d**) quantitative wear of the seal rings for the different tribopairs after 180 min of friction testing under fixed conditions of 500 N and 1000 r·min^−1^. Error bars in (**d**) represent the minimum-to-maximum range of three independent fixed-condition wear tests (n = 3).

Confocal microscopy was used to compare the worn surface morphology and two-dimensional profiles of the tribopairs, and the degree of wear was quantified using the wear rate of the rotating ring and the wear-step height of the stationary ring (Figure 7). The Graphite-MCD pair exhibited the lowest rotating-ring wear rate and stationary-ring step height (Figure 7d). In the SiC-SiC pair, both seal faces remained relatively flat and the profile curves showed only minor fluctuations. Slight wear marks were visible on the rotating ring, but the change in profile height was small on both faces (Figure 7a). This apparently limited material removal resulted from rapid adhesive interaction and severe mechanical interlocking between the SiC surfaces under high load in water, which terminated the test before substantial cumulative wear could develop. The Graphite-SiC pair showed strongly asymmetric wear (Figure 7b). A distinct annular wear zone and local debris were observed on the SiC rotating ring, accompanied by moderate profile fluctuations. The graphite stationary ring, by contrast, became markedly roughened and displayed irregular damage features, large profile fluctuations, and a pronounced reduction in surface height, indicating severe graphite removal. In the Graphite-MCD pair, the MCD rotating ring remained largely intact, with only localized wear traces and a small amount of debris. Its profile was generally stable, although isolated abrupt variations occurred at a few positions (Figure 7c). The graphite stationary ring developed a relatively continuous and uniform annular wear band, but both the profile fluctuation and height loss were much smaller than those of the graphite ring paired with SiC. Thus, replacing SiC with polished MCD effectively reduced wear of the graphite counterface. At 500 N, 1000 r·min^−1^, and 180 min, the rotating-ring wear rates of the SiC-SiC, Graphite-SiC, and Graphite-MCD pairs were 8.3 × 10^−10^, 9.5 × 10^−10^, and 0.15 × 10^−10^ mm^3^·N^−1^·m^−1^, respectively (Figure S2). The corresponding stationary-ring wear-step heights were 64, 689, and 63 μm (Figure S3). The Graphite-MCD rotating ring therefore exhibited a substantially lower wear rate than the other two rotating rings, while its stationary-ring wear height was comparable to that of SiC-SiC and far below that of Graphite-SiC. Taken together, the surface morphology, profiles, and quantitative results show that graphite experienced the most severe material removal when paired with SiC, consistent with pronounced abrasive wear [13,14,26]. The Graphite-MCD pair maintained low wear on both seal faces.

**Figure 8:**
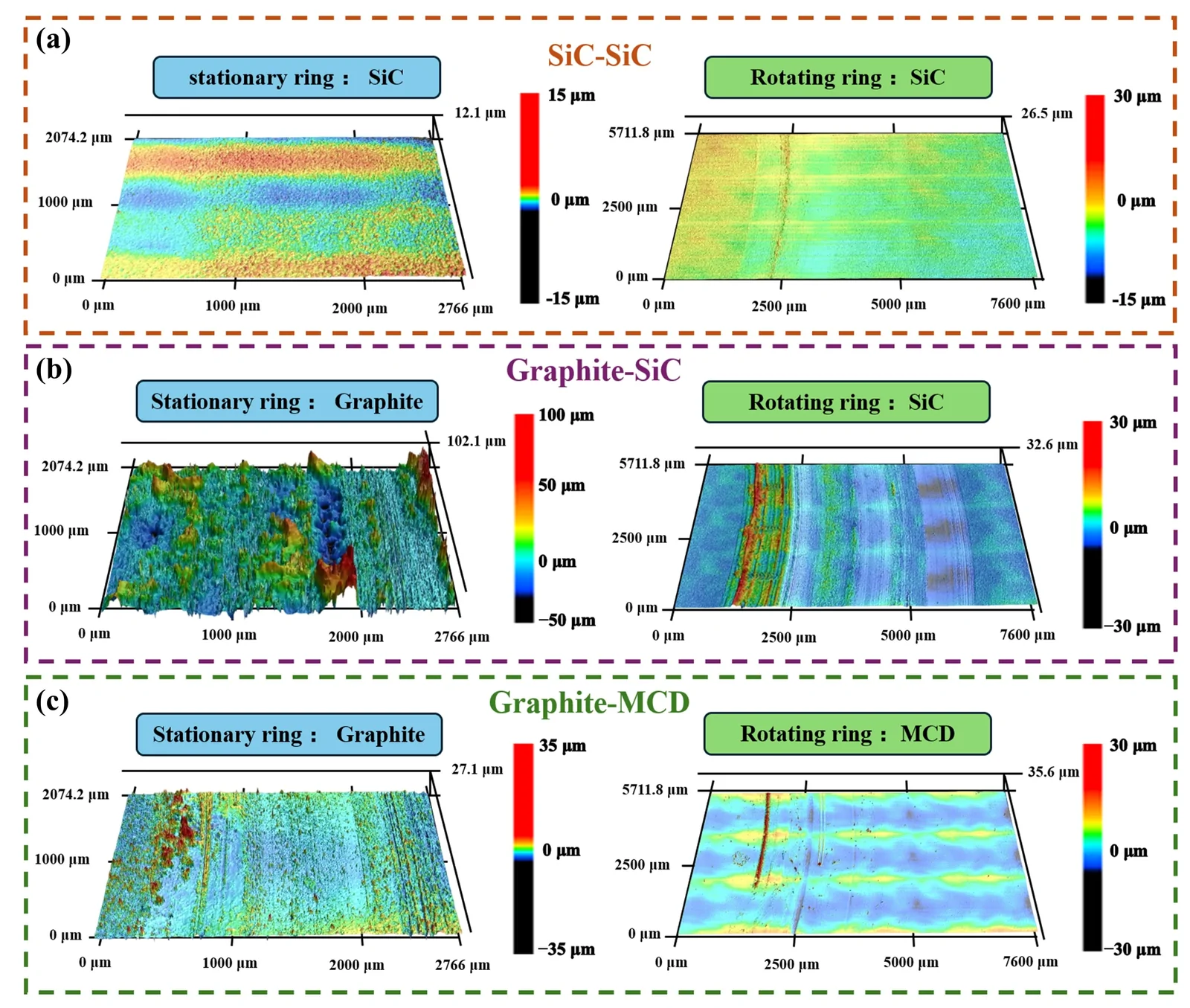
Three-dimensional wear morphologies of the rotating and stationary seal rings for the different seal-face tribopairs after the limiting tribological tests: (**a**) SiC–SiC, (**b**) Graphite–SiC, and (**c**) Graphite–MCD.

Three-dimensional confocal topographies were further analyzed to identify material-removal behavior under high-load water lubrication (Figure 8 and Figure S4). For the SiC-SiC pair, the worn surfaces of both rings were relatively flat, with only slight band-like undulations and a few shallow grooves. No obvious deep pits, spallation, or sharp protrusions were observed. The small topographic variation indicates limited material removal, although, as noted above, this pair failed rapidly through interfacial adhesion rather than sustained low-wear operation (Figure 8a). The Graphite-SiC pair displayed a markedly roughened graphite stationary ring containing numerous irregular protrusions, deep depressions, and local spallation, with severe peak-to-valley variation. The SiC rotating ring exhibited continuous circumferential grooves and band-like wear tracks. The height maps therefore reveal alternating high and low regions on the graphite surface and groove-dominated damage on SiC. These features are characteristic of abrasive plowing and local graphite spallation and indicate highly nonuniform material removal [1,27] (Figure 8b). When SiC was replaced by MCD, roughening and local spallation of the graphite ring were strongly suppressed. The graphite wear track consisted mainly of relatively uniform shallow grooves and small local protrusions, without the extensive deep pits and severe relief observed for Graphite-SiC. The MCD rotating ring retained high surface integrity and showed primarily continuous, wave-like undulations and a small number of narrow, relatively deep linear scratches. Height changes on both surfaces were more continuous, and anomalous peaks and depressions were markedly reduced, indicating more uniform and stable material removal (Figure 8c). The high hardness, low roughness, and dimensional stability of polished MCD reduced local stress concentration and abrasive plowing, while graphite provided a low-shear interface [28,29]. Their synergy accounts for the more uniform wear morphology and improved surface integrity of the Graphite-MCD pair.

### 3.4. Wear and Tribochemical Mechanisms

**Figure 9:**
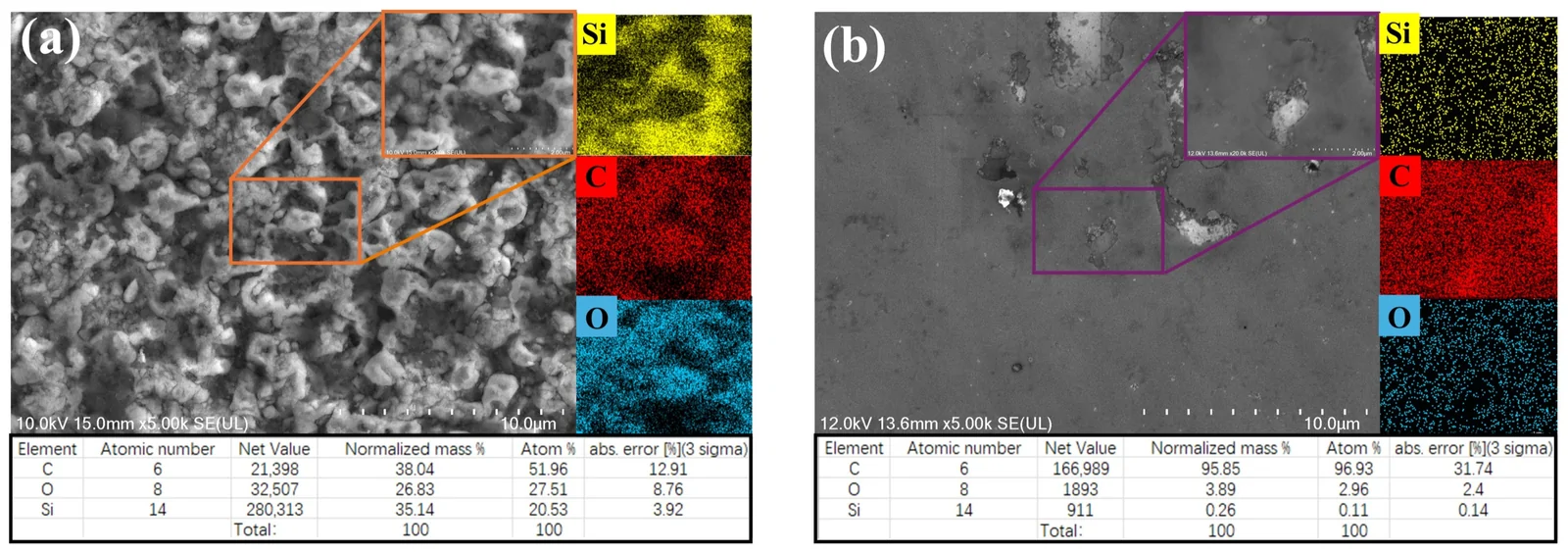
SEM images and EDS spectra of the wear tracks on the rotating rings for the different tribopairs after the limiting tribological tests: (**a**) Graphite–SiC and (**b**) Graphite–MCD.

SEM and EDS mapping were used to clarify the wear mechanisms of the Graphite-SiC and Graphite-MCD pairs (Figure 9). After sliding against graphite, the SiC wear track was rough and covered with abundant irregular particulate and agglomerated debris, together with local spallation and debris accumulation (Figure 9a). In addition to a high carbon content, pronounced Si and O signals were detected, and their spatial distributions were strongly correlated. This result indicates oxidation and brittle fragmentation of SiC with the formation of silicon-containing oxides [29]. Once introduced into the contact, these oxide products mixed with graphite and covered the sliding interface. The resulting mixed layer provided effective lubrication and substantially reduced the friction coefficient [30,31]. In contrast, the MCD wear track in the Graphite-MCD pair was comparatively smooth and contained only a few isolated protrusions or adherent features. Extensive debris accumulation, pronounced plowing grooves, and severe spallation were absent (Figure 9b). Carbon dominated the EDS maps, while Si and O were present only at low levels and showed no clear enrichment. The smooth morphology and absence of substantial hard-particle accumulation indicate that severe abrasive plowing and cutting did not occur. Given the high hardness and relative chemical inertness of MCD, the diamond surface underwent no obvious wear under these conditions.

**Figure 10:**
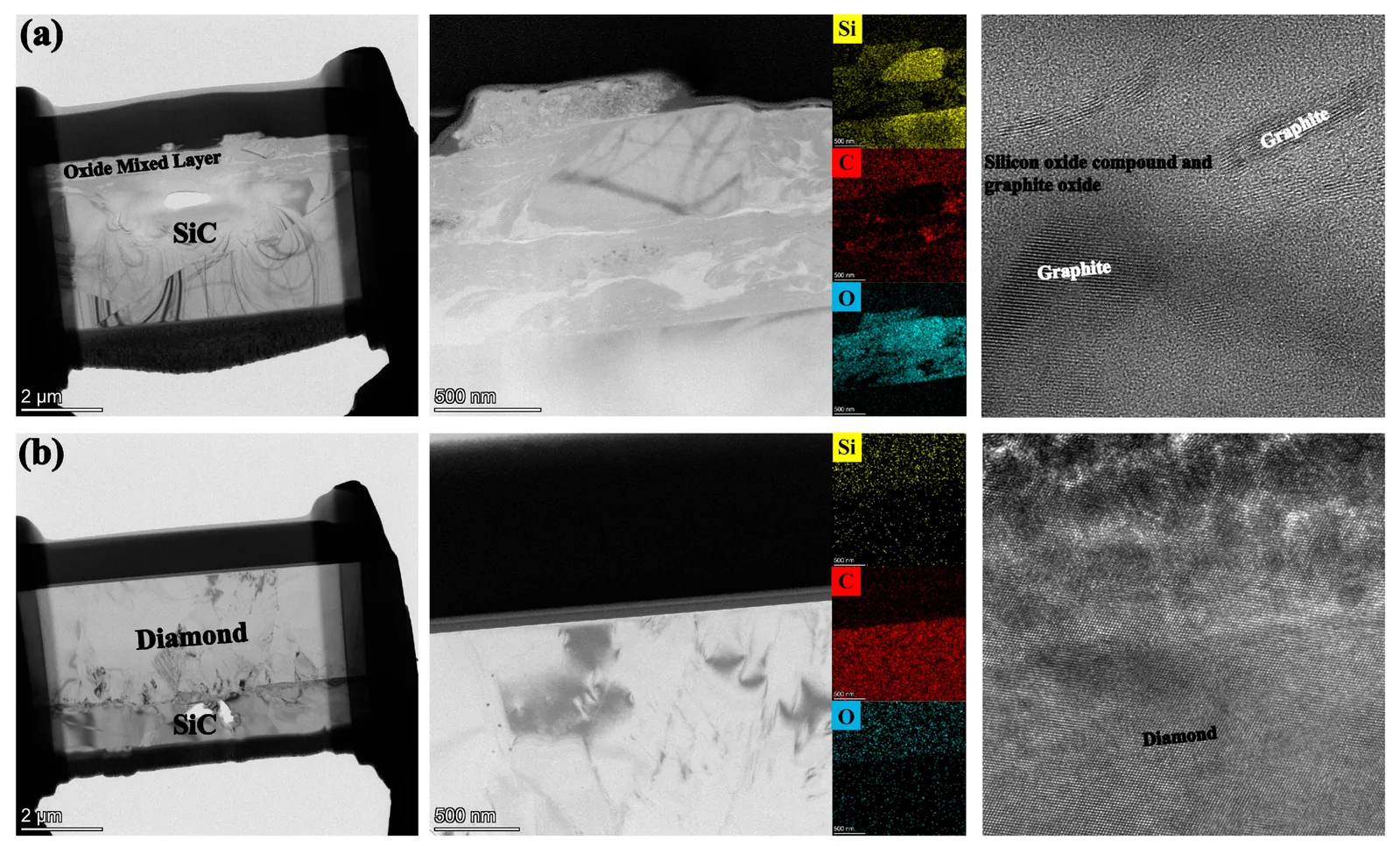
TEM images and EDS spectra of the wear tracks on the rotating rings for the different tribopairs after the limiting tribological tests: (**a**) Graphite–SiC and (**b**) Graphite–MCD.

TEM and EDS mapping revealed distinct near-surface structural evolution for the two hard counterfaces (Figure 10). In the Graphite-SiC pair, a continuous interfacial layer developed on the worn SiC surface, and SiC fragments were observed (Figure 10a). These fragments confirm brittle fracture of SiC during sliding and provide hard third-body particles that can intensify abrasion. Si, C, and O were simultaneously enriched in the near-surface region, evidencing substantial elemental redistribution and the presence of abundant SiO_x_. Lattice fringes in the high-resolution images further revealed graphitized carbon. The near-surface tribolayer was therefore composed of graphite-derived carbon mixed with SiO_x_ [32]. No continuous interfacial layer formed on the MCD near-surface region in the Graphite-MCD pair (Figure 10b). Carbon was distributed uniformly, the high-magnification images showed an ordered diamond lattice, and no abrasive particles were observed at the surface. Thus, the two hard counterfaces exhibited fundamentally different structural stability during sliding: Graphite-SiC developed a continuous amorphous near-surface layer accompanied by elemental redistribution, whereas Graphite-MCD preserved the integrity of the diamond lattice and a stable interfacial microstructure. This higher structural stability is a key reason for the stable wear mode of the Graphite-MCD pair.

**Figure 11:**
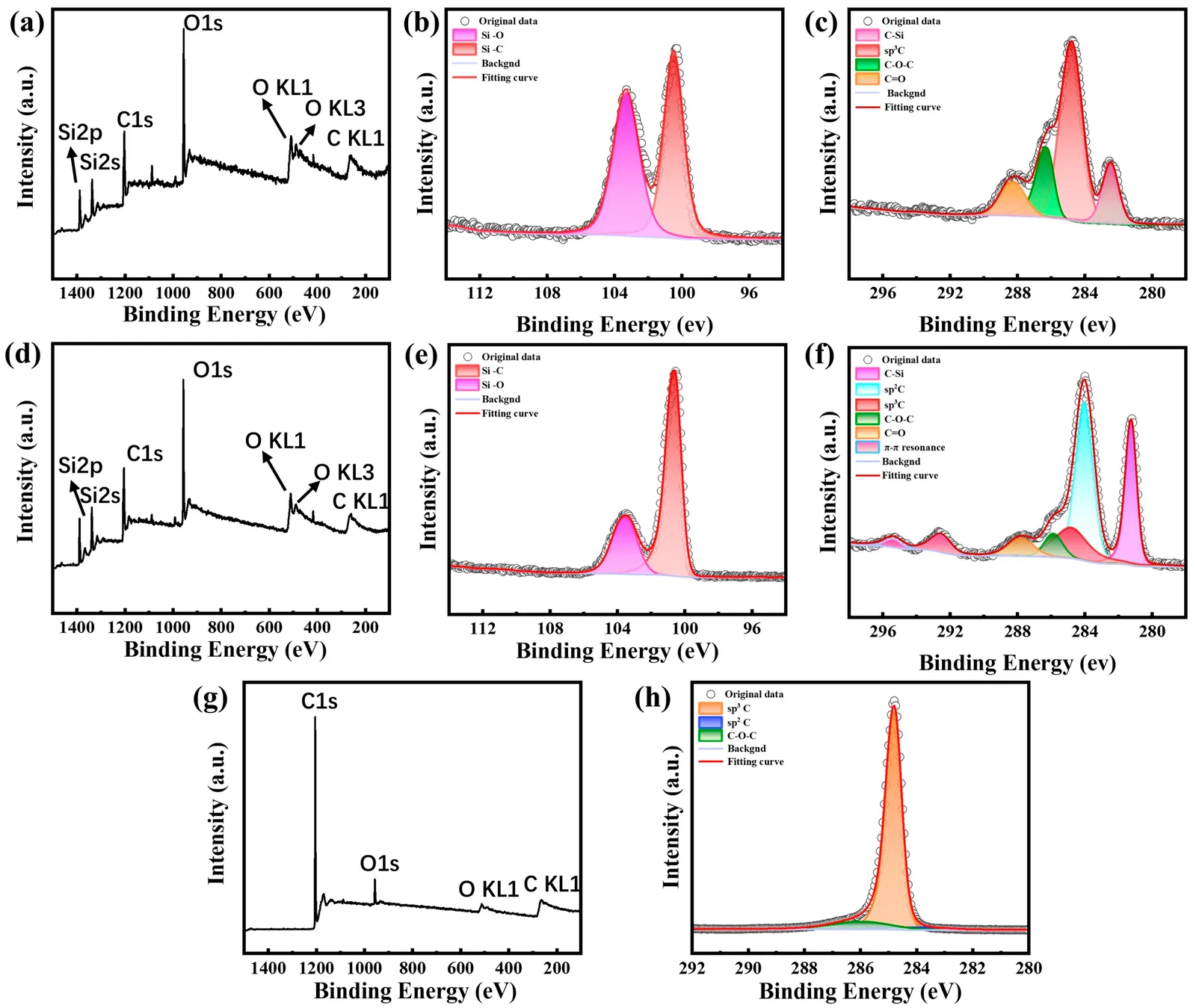
XPS results of the rotating rings for the different tribopairs after the limiting tribological tests: (**a**–**c**) SiC-SiC, (**d**–**f**) Graphite-SiC, and (**g**,**h**) Graphite-MCD.

XPS was employed to determine how the hard counterface controls tribochemical evolution (Figure 11). For the SiC-SiC pair, the survey spectrum contained prominent Si 2p, O 1s, and C 1s signals, indicating that the sliding interface was composed mainly of Si, O, and C (Figure 11a). Deconvolution of Si 2p produced components at approximately 100.6 eV (Si-C) and 103.5 eV (Si-O) [33] (Figure 11b). The Si-C signal originated from the exposed SiC near-surface region, whereas Si-O indicated oxidation of SiC under water-lubricated sliding and partial conversion of near-surface Si-C bonds into oxidized silicon species. The C 1s spectrum of the SiC-SiC wear track contained C-Si (approximately 282.0 eV), C-C/sp^3^ C (284.8 eV), C-O-C (286.0 eV), and C=O (288.5 eV) components (Figure 11c). The low-binding-energy C-Si peak confirms exposure of the SiC substrate. The oxygen-containing carbon species demonstrate that carbon in SiC underwent oxidation and formed oxygenated functional groups, whereas the sp^3^-carbon component may be associated with friction-induced Si-C bond rupture followed by carbon-structure reconstruction [34]. The Graphite-SiC wear track also exhibited Si 2p, O 1s, and C 1s signals. Its Si 2p spectrum contained Si-C and Si-O components at approximately 100.6 and 103.5 eV, respectively (Figure 11e), but the Si-O intensity was lower than that of SiC-SiC. The introduction of graphite therefore mitigated SiC oxidation, likely because a carbon-rich lubricating film covered the interface and reduced direct contact between the aqueous reactive species and the SiC substrate. The C 1s spectrum was fitted with C-Si (approximately 282.0 eV), C=C/sp^2^ C (284.0 eV), C-C/sp^3^ C (284.8 eV), C-O-C (286.0 eV), O-C=O (288.5 eV), and a π-π* resonance at 292–294 eV (Figure 11f). The dominant sp^2^-carbon peak and the π-π* feature confirm retention of a highly graphitized carbon structure. The oxygen-containing carbon components indicate concurrent oxidation of graphite and/or carbon from SiC. Graphite-SiC thus underwent mechanical shear, structural reconstruction, and pronounced tribochemical reactions, producing a complex reaction layer composed of Si-O species, oxygenated carbon, and graphitized carbon. By comparison, the XPS survey spectrum of the MCD wear track after sliding was dominated by C 1s, with only a weak O 1s signal (Figure 11g). The high-resolution C 1s spectrum consisted mainly of sp^3^ carbon (Figure 11h), demonstrating that the coating largely retained its diamond-like bonding structure. The MCD near-surface region therefore exhibited higher chemical stability and effectively suppressed friction-induced interfacial reactions [30]. Overall, SiC-SiC underwent Si-C oxidation and carbon reconstruction; Graphite-SiC generated a graphite/Si-O/oxygenated-carbon triboreaction layer; and Graphite-MCD retained a predominantly sp^3^-bonded diamond surface with only limited reconstruction and oxidation. The polished MCD coating consequently provided superior chemical inertness and interfacial stability.

### 3.5. Comparative Analysis of Friction Mechanisms in Different Tribopairs

**Figure 12:**
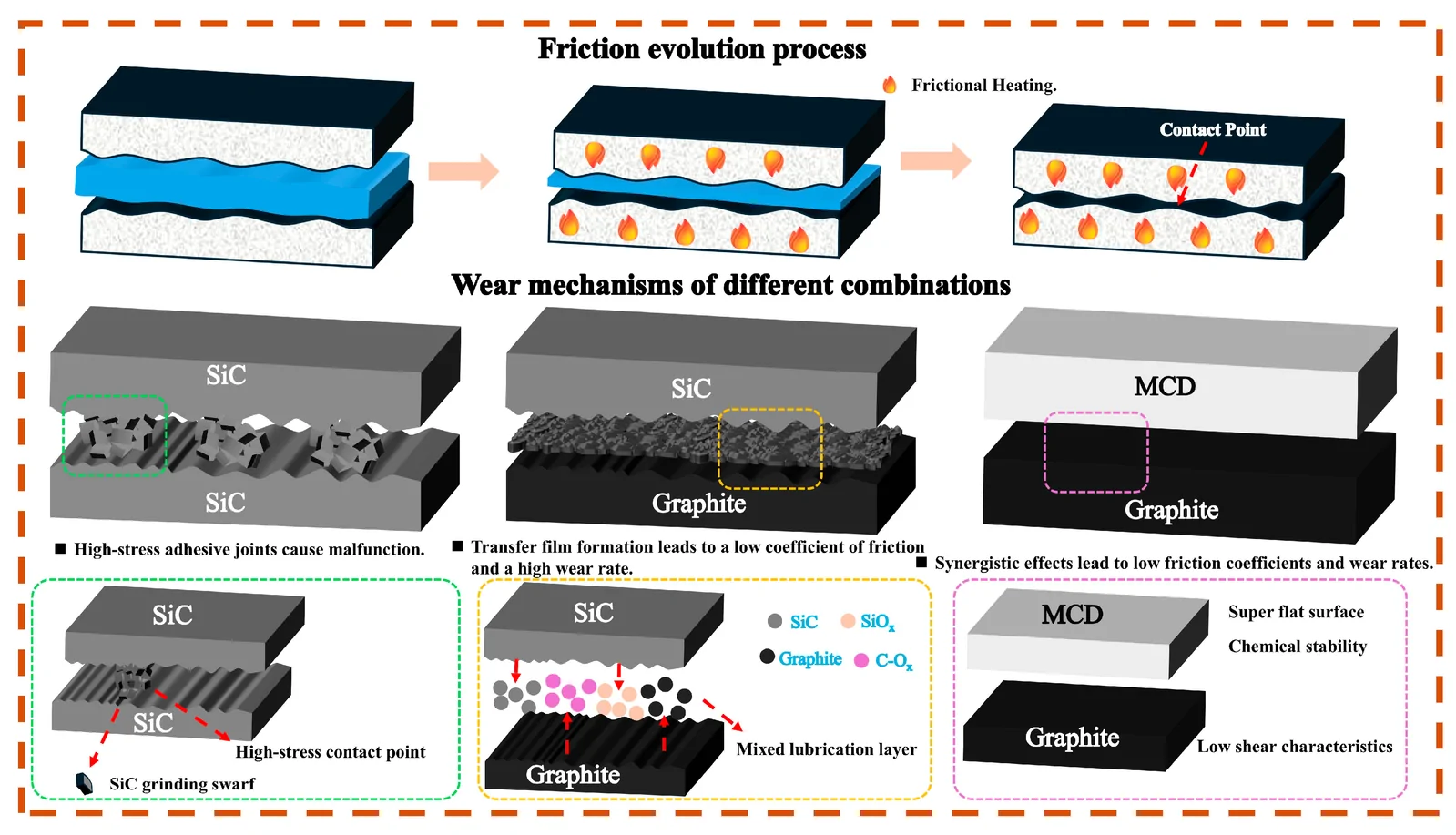
Schematic illustration of the interfacial friction and wear mechanisms of SiC-SiC, Graphite-SiC, and Graphite-MCD under water lubrication.

Based on the preceding analyses, Figure 12 summarizes the proposed interfacial evolution of the three seal-face tribopairs under water lubrication. At the beginning of sliding, a water film separates the mating faces and the seal operates predominantly in a hydrodynamic lubrication regime [19,35]. As the rotational speed increases, hydrodynamic pressure, frictional heating, face deformation, and interfacial film evolution all intensify. When the capacity to generate a fluid film can no longer compensate for local closure caused by thermomechanical deformation and surface damage, the interface shifts from stable mixed lubrication to unstable local contact, eventually producing torque fluctuations or leakage. At this stage, the material combination controls the subsequent wear mechanism and therefore the limiting sealing and tribological performance. For SiC-SiC, breakdown of the water film brings asperities on the two hard ceramic surfaces into direct contact. Because no low-shear lubricating phase is present, local adhesive junctions readily form in high-stress contact regions. Continued shear destabilizes these junctions, leading to adhesive seizure, abnormal torque fluctuations, and premature seal failure. The small measured material loss therefore does not indicate benign operation; it reflects rapid failure caused by mechanical interlocking and adhesion. For Graphite-SiC, graphite continuously transfers to the SiC surface and forms a carbon-rich transfer film. At the same time, detached SiC fragments react tribochemically with water under frictional heat and pressure to produce silicon-oxide-containing products. The graphite transfer film and oxide-rich layer jointly reduce interfacial shear strength and suppress adhesive wear. However, brittle fracture of SiC repeatedly generates and disrupts the interfacial layer under cyclic loading. The resulting coarse SiC debris acts as a third body and promotes abrasive wear, particularly on the graphite face. Graphite-SiC therefore alleviates adhesive damage relative to SiC-SiC, but abrasive wear driven by brittle SiC fragmentation becomes the dominant failure mode. For Graphite-MCD, the polished MCD coating combines high hardness, strong chemical stability, low roughness, and excellent dimensional stability, thereby suppressing adhesion, tribochemical reaction, and surface fracture. Graphite simultaneously acts as a stable, low-shear solid lubricant throughout sliding. Because the polished MCD surface generates almost no hard wear debris, third-body abrasion is strongly inhibited. The contact can therefore retain both stable lubrication and reliable mechanical support without transitioning to severe adhesion- or abrasion-dominated damage. This synergy lowers both adhesive and abrasive wear and accounts for the superior sealing stability, lowest wear rate, and highest limiting PcV of the Graphite-MCD pair.

## 4. Conclusions

A novel Graphite-MCD soft–hard seal-face tribopair was developed to address insufficient interfacial load capacity and wear-induced failure of mechanical seals in high-pressure water environments. The structural characteristics of the polished MCD coating, the sealing performance of different tribopairs, and their interfacial failure mechanisms were systematically investigated. The main conclusions are as follows:

1. The seal-face material combination strongly controlled the limiting load capacity. Because SiC-SiC lacked an effective low-shear lubricating component, breakdown of the water film caused direct ceramic–ceramic contact, local adhesion, and frictional instability, resulting in the lowest limiting PcV of 3.40 MPa·m·s^−1^. Graphite reduced interfacial shear resistance through its layered structure and transfer-film formation, increasing the limiting PcV of Graphite-SiC to 50.19 MPa·m·s^−1^; however, graphite removal and third-body wear persisted. Graphite-MCD exhibited the highest limiting PcV, 90.73 MPa·m·s^−1^, demonstrating that the polished MCD coating substantially improved the operational stability of the soft–hard pair under high-load and high-speed water lubrication.
2. The hard counterface produced distinct wear-evolution pathways. SiC-SiC failed primarily through adhesive instability and mechanical interlocking. Although cumulative material removal was limited, stable operation could not be sustained. In Graphite-SiC, graphite and friction-generated silicon oxides formed the principal lubricating film, but brittle fracture of SiC generated hard debris that caused severe graphite removal and abrasive wear. In Graphite-MCD, the MCD surface retained high integrity with only limited localized damage, while graphite provided a low-shear interface, enabling stable operation with low wear.
3. The chemical stability of the hard material determined the long-term evolution of the sliding interface. SiC participated in pronounced tribochemical reactions. SiC-SiC formed Si-O bonds, oxygenated carbon species, and reconstructed carbon, whereas Graphite-SiC developed a complex reaction layer containing graphitized carbon, Si-O species, and oxygenated carbon. Although this layer reduced shear resistance, it also promoted third-body formation. In Graphite-MCD, the surface remained predominantly sp^3^-bonded with only minor oxidation, reflecting superior chemical inertness and interfacial stability.
4. Improving the limiting PcV of a mechanical seal does not depend solely on lowering the friction coefficient; it requires synergistic stability of the lubricating and load-bearing interfaces. In the Graphite-MCD pair, graphite provides a low-shear lubricating interface, while polished MCD provides a stiff load-bearing interface and preserves both structural and chemical stability. This combination unifies low friction, high load capacity, and low wear. The resulting design principle—low-shear lubrication, high-stiffness load support, and interfacial stability—offers a mechanistic framework for seal materials used in deep-sea mechanical seals, seawater pumps, and high-speed water-lubricated bearings.

## Data Availability

The original contributions presented in this study are included in the article/Supplementary Materials. Further inquiries can be directed to the corresponding authors.

## Supplementary Materials

The following supporting information can be downloaded at: https://www.mdpi.com/article/10.3390/ma19183810/s1, Figure S1: The limiting PcV values obtained from tests with three different material pairings: (a) SiC–SiC, (b) Graphite–SiC, and (c) Graphite–MCD. Figure S2: Cross-sectional areas of wear tracks on rotating rings with different tribopairs at different locations under identical test conditions (a–c) SiC–SiC, (d–f) Graphite–SiC, (g–i) Graphite–MCD. Figure S3: Two-dimensional wear track profiles of the rotating rings and wear heights of the stationary rings before and after friction tests. (a,d) SiC–SiC, (b,e) Graphite–SiC, and (c,f) Graphite–MCD. Figure S4: Initial surface morphologies of graphite and SiC materials before testing: (a) Graphite, (b) SiC.
