# Supplementary material for: Limiting PcV and Tribological Performance of Mechanical Seals with Hard–Hard and Soft–Hard Tribopairs Under Water Lubrication
Source: Materials (Basel). 2026 Sep 8;19(18):3810. doi: 10.3390/ma19183810 (PMC13608359; doi:10.3390/ma19183810)
Supplement: Supplementary file 1 [file materials-19-03810-s001.zip › materials-4512476-supplementary.pdf]

1. The normal load was fixed at 2000 N. The initial rotational speed was set to 200 $r \cdot min^{-1}$ and increased to 10,000 $r \cdot min^{-1}$ at a constant acceleration rate of 100 $r \cdot min^{-1}$, under which the limiting PcV value was determined.

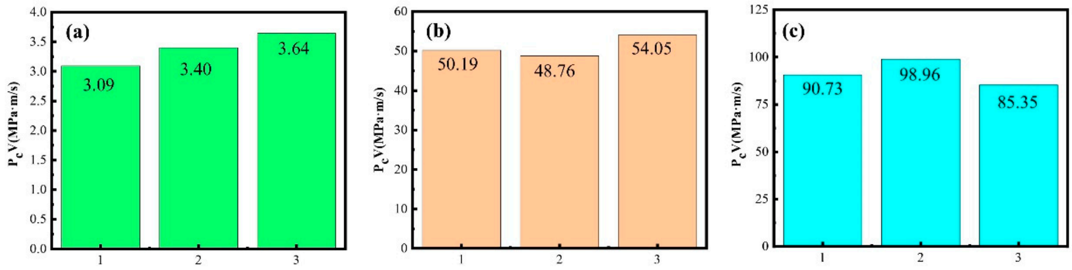

Figure S1. The limiting PcV values obtained from tests with three different material pairings: (a) SiC–SiC, (b) Graphite–SiC, and (c) Graphite–MCD.

2. Two-dimensional wear area of the rotating ring after running for 180 min under a constant load of 500 N and a constant rotational speed of 1000 $r \cdot min^{-1}$.

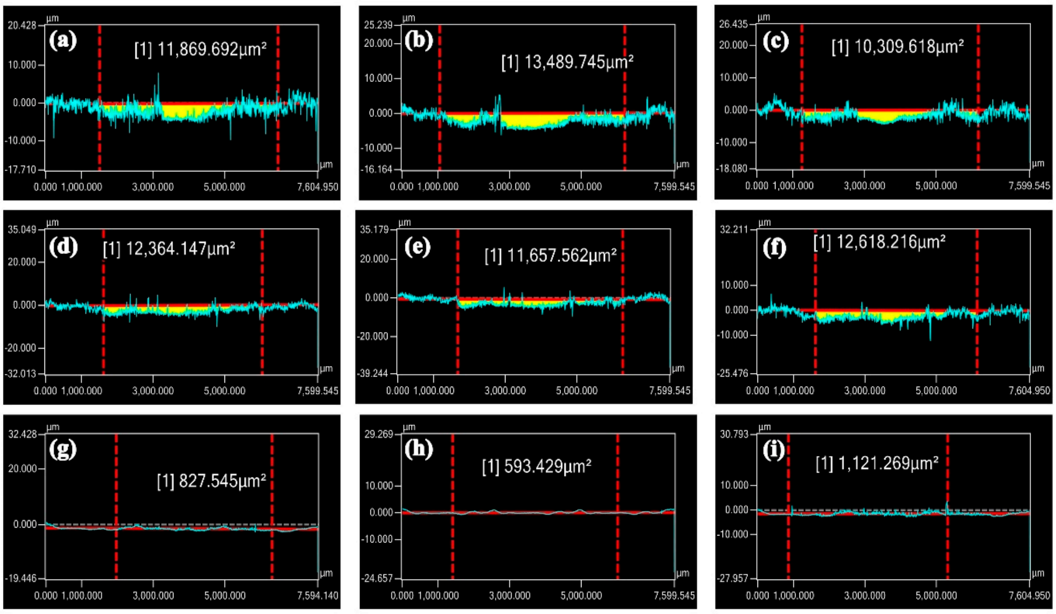

Figure S2. Cross-sectional areas of wear tracks on rotating rings with different tribopairs at different locations under identical test conditions (a-c) SiC–SiC, (d-f) Graphite–SiC, (g-i) Graphite–MCD.

3.Two-dimensional wear profiles of the rotating and stationary rings after running for 180 min under a constant load of 500 N and a constant rotational speed of 1000 $r \cdot min^{-1}$.

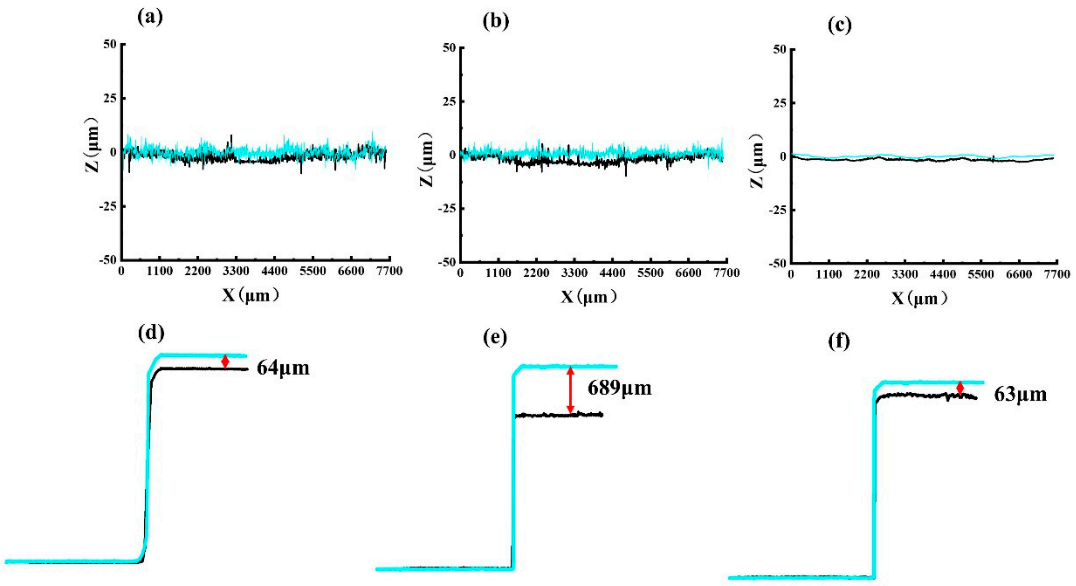

Figure S3. Two-dimensional wear track profiles of the rotating rings and wear heights of the stationary rings before and after friction tests. (a)、(d) SiC–SiC, (b)、(e) Graphite–SiC, and (c)、(f) Graphite–MCD.

4. 3D Surface Morphology of the Initial Graphite Ring and Silicon Carbide Ring.

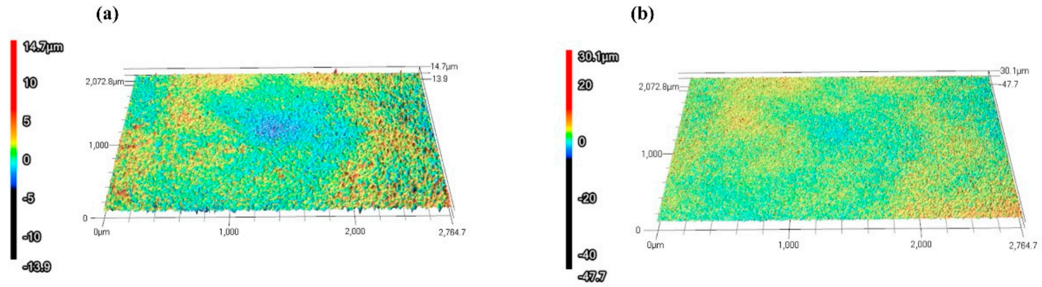

Figure S4. Initial surface morphologies of graphite and SiC materials before testing: (a) Graphite, (b) SiC.
